# Adiposity indices and their higher predictive value for new-onset hypertension in metabolically healthy young women: findings from a population-based prospective cohort study

**DOI:** 10.1186/s12872-024-03817-y

**Published:** 2024-03-12

**Authors:** Rizki Amalia Gumilang, Yen-Chun Fan, Shang-Hao Wu, Chyi-Huey Bai

**Affiliations:** 1 International Master/Ph.D. Program in Medicine, College of Medicine, Taipei Medical University, Taipei, Taiwan; 2 Faculty of Medicine, Public Health, and Nursing/Academic Hospital, Universitas Gadjah Mada, Yogyakarta, Indonesia; 3 Department of Public Health, College of Medicine, Taipei Medical University, Taipei, Taiwan; 4 School of Public Health, College of Public Health, Taipei Medical University, Taipei, Taiwan; 5 College of Nursing, National Taipei University of Nursing and Health Sciences, Taipei, Taiwan; 6 Department of Allied Health Education and Digital Learning, National Taipei University of Nursing and Health Sciences, Taipei, Taiwan

**Keywords:** Metabolically healthy, Taiwan biobank, Adiposity index, Body mass index, Waist circumference, Waist-to-hip ratio, Waist-to-height ratio, New-onset hypertension

## Abstract

**Background and aims:**

The present study aimed to investigate the predictive ability of selected adiposity indices, such as body mass index (BMI), waist-to-hip ratio (WHR), waist circumference (WC), and waist-to-height ratio (WHtR), for new-onset hypertension in metabolically healthy Taiwanese adults. The study also sought to establish sex-specific cutoff points for these indices and to analyze the risk of new-onset hypertension, taking into account sex and age.

**Methods:**

This prospective cohort study utilized the Taiwan Biobank database to examine metabolically healthy participants aged between 20 and 65 at baseline. Four adiposity indices, namely BMI, WHR, WC, and WHtR, were calculated and used to predict new-onset hypertension over 4 years. Receiver operating characteristics (ROCs) and areas under the curve (AUCs) were used to evaluate the effectiveness of the parameters in predicting new-onset hypertension over 4 years. Sex-specific cutoff points were identified and used to assess the risk of new-onset hypertension.

**Results:**

This study analyzed 13,375 participants over 4.28 years. The incidence of new-onset hypertension was 17.65%. The new-onset rate of hypertension was 34.39% in men and 65.61% in women. Adiposity indices effectively predict new-onset hypertension, with WHtR having the highest predictive value (i.e., AUC) for both sexes. The classification of participants into low and high categories for each adiposity index was based on sex-specific cutoff points, and the risk of new-onset hypertension was assessed according to sex and age. This study found that high adiposity indices predicted a significantly higher risk of new-onset hypertension in metabolically healthy adults. The risk was equal for both sexes. Young women had a higher risk of new-onset hypertension than middle-aged women when they were further categorized. All risk ratios of the indices in young women were over two-fold and significant.

**Conclusion:**

According to the sex-specific cutoff point, high adiposity indices had a higher predictive value for new-onset hypertension in metabolically healthy Taiwanese young women.

**Supplementary Information:**

The online version contains supplementary material available at 10.1186/s12872-024-03817-y.

## Introduction

Hypertension is a significant contributor to cardiovascular mortality and morbidity, responsible for approximately half of such incidence [1]. Early screening and prevention programs can effectively reduce the burden of hypertension, which is a preventable cardiovascular risk factor [2]. The prevalence of hypertension varies by age, sex, and ethnicity, with Asia, particularly Southeast, South, and East Asia, having a high prevalence of the condition [3, 4]. The prevalence of hypertension in Taiwan reached 20.8%, as reported by the National Health and Nutrition Examination Survey (NHANES), conducted from 2013 to 2016 [5].

Excess adiposity is a significant risk factor for hypertension, and weight reduction is often included in blood pressure-lowering treatment [6–8]. In assessing excess adiposity, imaging modalities provide detailed information on the composition of excess adiposity. However, anthropometric adiposity indices are still preferred in clinical practice because of their convenience [9]. Clinical practice employs various adiposity indices, including the body mass index (BMI), waist-to-hip ratio (WHR), waist circumference (WC), and waist-to-height ratio (WHtR) [10, 11]. These parameters have been established as predictors of cardiometabolic risk, specifically hypertension [12, 13].

Adiposity can induce a rise in blood pressure without prior metabolic abnormalities through neurohumoral and renal mechanisms [14]. The mechanism of adiposity-induced hypertension also differed among sexes, mainly due to differences in hormonal regulation, sympathetic nervous system, and renin–angiotensin–aldosterone regulation [15, 16]. Sex should be considered when predicting hypertension through excess adiposity.

Additionally, adiposity's crucial role in identifying individuals who transition from metabolically healthy to metabolically unhealthy has also been highlighted [17–20]. The accumulation of adipose tissue, especially visceral fat, is often the first manifestation of metabolic disorders. In a 5-year follow-up study, researchers found that the younger the age at which adults became overweight, the higher their risk of developing high blood pressure. The group with the highest relative risk was those who became overweight between the ages of 18 and 39, with overweight being the only risk factor for these subjects [21]. This shows the importance of detecting early obesity in metabolically healthy individuals. Consequently, the impact of excess adiposity on predicting hypertension in metabolically healthy individuals requires further investigation, particularly considering age and sex.

The aims of this study were twofold: (1) to investigate the predictive power of selected adiposity indices, including BMI, WHR, WC, and WHtR, and to establish sex-specific cutoff points for these indices about new-onset hypertension among metabolically healthy Taiwanese adults; and (2) to evaluate the risk of new-onset hypertension in metabolically healthy Taiwanese adults by utilizing these adiposity indices, taking into account age and sex.

## Materials and methods

### Study design and population

This prospective cohort study included participants selected from the Taiwan Biobank (TWB) database. The TWB is a significant genomic database for Chinese populations, containing extensive demographic and health-related survey data, physical measurements, biochemical data, and genomic data, all aimed at enhancing the capability of clinicians to prescribe personalized and precise medication in Taiwan [22, 23]. All patients submit written informed consent before being enrolled in the TWB [24]. The Taipei Medical University Institutional Review Board (number N202104112) approved the study after receiving clearance.

**Fig. 1:**
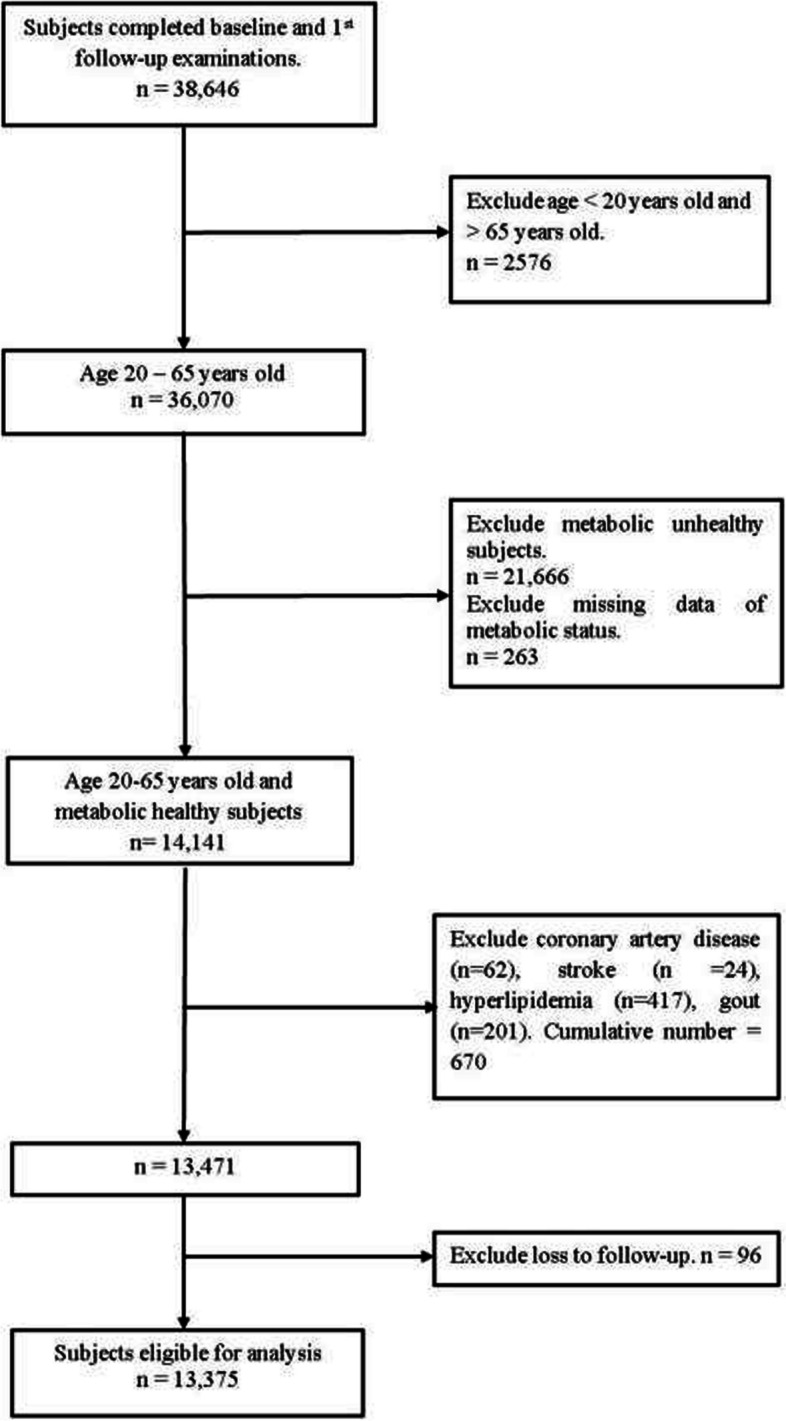
Recruitments of the study participants

In this study, the population is the individuals with two interviews, those between 20 and 65 years of age, who were metabolically healthy at baseline. Participants with a history of self-reported coronary artery disease, stroke, hyperlipidemia, hypertension, or gout at baseline were excluded. Of the 14,141 participants who fulfilled the inclusion criteria, 62 reported a history of coronary artery disease, 417 had hyperlipidemia, 24 had a history of stroke, 201 had gout, and none had hypertension. Moreover, 96 subjects who had missing data for blood pressure measurements and/or self-reported hypertension status during the first follow-up (loss to follow-up) were excluded from the study. A total of 13,375 participants were included in this study (Fig. 1).

### Data collection

Questionnaires, blood samples, and physical examinations were collected. Age, sex, menopausal status, self-reported smoking status, and alcohol consumption were recorded. Smoking status was divided into three categories: never/occasional, quitting, and still smoking. Alcohol consumption was divided into three categories: never/occasional drinking, quitting drinking, and drinking.

Blood pressure measurements were performed on the right upper arm, with the patient sitting, using a sphygmomanometer after a 5-min rest. Average systolic blood pressure (SBP) and diastolic blood pressure (DBP) were used in the analysis. A calibrated body scale measured the weight (kg) and height (cm). Waist circumference (WC; cm) was measured around the abdomen immediately superior to the iliac crest. Hip circumference (HC; cm) was the largest circumference across the buttock.

Fasting blood samples were collected from the antecubital vein. Fasting plasma glucose (FPG; mg/dL), triglyceride (TG; mg/dL), total cholesterol (TC; mg/dL), and high-density lipoprotein cholesterol (HDL-C; mg/dL) levels were measured using an autoanalyzer (COBAS Integra 400, Roche Diagnostics, Mannheim, Germany).

### Definitions of metabolic health

In our study, four metabolic indicators were considered: (1) SBP ≥ 130 mmHg or DBP ≥ 80 mmHg; (2) elevated fasting glucose levels (≥ 100 mg/dL); (3) raised triglycerides (≥ 150 mg/dL); and (4) reduced HDL-C (< 40 mg/dL in men and < 50 mg/dL in women) [25, 26]. A metabolically healthy individual has none of these four metabolic indicators [27].

### Determining adiposity indices

BMI, WHR, and WHtR were calculated using the following formulas [28, 29]:

$$\begin{array}{ll}\mathrm{BMI }=\mathrm{weight}\ ({\text{kg}})/\mathrm{height}\ ({\text{m}})^2\\ \mathrm{WHR }=\mathrm {WC}\ ({\text{cm}})/\mathrm{HC}\ ({\text{cm}})\\ \mathrm{WHtR }=\mathrm{ WC }\ ({\text{cm}})/\mathrm{height}\ ({\text{cm}})\end{array}$$

### Outcome definition

The follow-up duration was calculated from baseline to ^the first^ follow-up. The outcome was the presence of new-onset hypertension, which is defined as SBP ≥ 130 mmHg or DBP ≥ 80 mmHg or self-reported hypertension in the ^first^ follow-up (the criteria of hypertension of the 2022 Guidelines of the Taiwan Society of Cardiology or the Taiwan Hypertension Society for the Management of Hypertension) [26].

### Statistical analysis

All statistical computations were performed using the SAS software (version 9.4; SAS Institute, Cary, NC, USA). Continuous variables are presented as means ± standard deviations and were tested using *the t-test*. Categorical variables are presented as numbers (percentages) and tested using the chi-square test.

Receiver operating characteristics (ROCs) and areas under the curve (AUCs) were used to assess the classification fitness of each index for hypertension. The ROC curves of the four indices were compared using the Wald test. The cutoff points for each index were determined using the maximal Youden index (*J*), calculated as sensitivity + specificity − 1 [30]. We then divided the participants into lower and upper groups based on the cutoff value.

The risk ratios (RRs) of hypertension for the upper cutoff group were compared with those for the lower cutoff group using binomial logistic regression (PROC GENMOD statement in SAS). The hypertension models were stratified by sex and age. Two age groups were: a young-adult (20–45 years old) group and a middle-aged-adult (46–65 years old) group [31]. Forrest plots were generated using Medcalc Software to visualize the RRs.

## Results

### Baseline characteristics

Following the exclusion of ineligible participants, 13,375 were included in the analysis, as depicted in Fig. 1. The median follow-up period in participants was 4.28 years. The average age was 46.67 years, with women comprising 76.96% of the total population. There were 2631 cases (17.65%) of new-onset hypertension. The new-onset rate of hypertension was 34.39% in men and 65.61% in women. The mean values of the adiposity indices were 22.39 kg/m_2_ for BMI, 0.83 for WHR, 78.04 cm for WC, and 0.49 for WHtR. The distribution of these indices is shown in Figure S1 in the Supplementary Materials.

Participants with new-onset hypertension tended to have higher adiposity indices. Metabolic parameters such as FPG, TG, and TC were higher in patients with new-onset hypertension, and HDL-C was lower than in those without hypertension. Other risk factors appeared to differ significantly between the two groups (Table 1). As the population was dominated by women (76.76%), the baseline characteristics of the subjects were also assessed according to sex.

**Table 1:** Baseline characteristics of subjects according to new-onset hypertension and sex

	Total Population *n* = 13,375	New-onset hypertension in follow-up			Sex		
Variable		No *n* = 11,014	Yes *n* = 2361	*p*-value	Men *n* = 3082	Women *n* = 10,293	*p*-value
Age at baseline	46.67 ± 9.77	45.91 ± 9.64	50.22 ± 9.61	< .0001	45.64 ± 10.45	46.98 ± 9.54	< .0001
Age category				< .0001			< .0001
Young aged	6211(46.44)	5473(49.69)	738 (31.26)		1604(52.04)	4607(44.76)	
Middle aged	7164 (53.56)	5541(50.31)	1623(68.74)		1478(47.96)	5686(55.24)	
Sex				< .0001			
Men	3082 (23.04)	2270(20.61)	812(34.39)		NA	NA	
Women	10,293(76.96)	8744(79.39)	1549(65.61)		NA	NA	
FPG	88.68 ± 5.44	88.35 ± 5.40	90.24 ± 5.34	< .0001	90.67 ± 4.93	88.09 ± 5.44	< .0001
TG	74.27 ± 27.35	72.81 ± 26.90	81.08 ± 28.40	< .0001	82.61 ± 28.61	71.77 ± 26.46	< .0001
HDL-C	61.81 ± 11.39	62.26 ± 11.38	59.69 ± 11.21	< .0001	53.40 ± 9.86	64.32 ± 10.59	< .0001
TC	192.68 ± 32.13	191.9 ± 32.17	196.4 ± 31.69	< .0001	187.9 ± 30.87	194.1 ± 32.35	< .0001
SBP	106.63 ± 10.63	104.9 ± 10.17	114.8 ± 8.80	< .0001	110.6 ± 9.12	105.4 ± 10.76	< .0001
DBP	66.29 ± 7.14	65.26 ± 6.96	71.07 ± 5.89	< .0001	69.65 ± 6.21	65.28 ± 7.09	< .0001
BMI	22.39 ± 2.90	22.14 ± 2.81	23.51 ± 3.04	< .0001	23.41 ± 2.83	22.08 ± 2.85	< .0001
WHR	0.83 ± 0.06	0.83 ± 0.06	0.86 ± 0.06	< .0001	0.87 ± 0.05	0.82 ± 0.06	< .0001
WC	78.04 ± 8.25	77.32 ± 8.06	81.38 ± 8.29	< .0001	82.37 ± 7.63	76.74 ± 7.98	< .0001
WHtR	0.49 ± 0.05	0.48 ± 0.05	0.51 ± 0.05	< .0001	0.48 ± 0.05	0.49 ± 0.05	0.08
Current smoking Status				< .0001			< .0001
No/occasionally	10,998(88.11)	9200(89.22)	1798(82.86)		1608(60.61)	9390(95.53)	
Quit smoking	723 (5.79)	540(5.24)	183(8.43)		524(19.75)	199(2.02)	
Still smoking	761 (6.10)	572(5.55)	189(8.71)		521(19.64)	240(2.44)	
Regular exercise				< .0001			0.84
No	7980(59.73)	6677(60.69)	1303(55.21)		1836(59.57)	6144(59.77)	
Yes	5381 (40.27)	4324(39.31)	1057(44.79)		1246(40.43)	4135(40.23)	
Alcohol consumption				< .0001			< .0001
No/occasional	12,733(95.31)	10,544(95.85)	2189(92.75)		2691(87.48)	10,042(97.65)	
Quit drinking	179 (1.34)	141(1.28)	38(1.61)		111(3.61)	68(0.66)	
Still drinking	448(3.35)	315(2.86)	133(5.64)		274(8.91)	174(1.69)	
Menopause status				< .0001			NA
No	6284(61.08)	5603(64.10)	681(44.02)		NA	6284(61.08)	
Yes	4004(38.92)	3138(35.90)	866(55.98)		NA	4004(38.92)	

Categorical variables in number (%); continuous variables in mean± SD

*FPG* Fasting plasma glucose level, *TG* Triglyceride level, *HDL-C* High-density lipoprotein cholesterol level, *TC* Total cholesterol level, *SBP *Systolic blood pressure, *DBP* Diastolic blood pressure, *BMI* Body mass index, *WHR* Waist-to-hip ratio, *WC* Waist circumference, *WHtR *Waist-to-height ratio

The mean values of adiposity indices were found to be higher in men, except for WHtR, which was similar between men and women (0.48 vs. 0.49, *p* = 0.08). Men demonstrated higher levels of FPG and TG and lower levels of TC and HDL-C than women. Additionally, men had higher baseline SBP and DBP values than women (SBP 110.6 vs. 105.4 mmHg, *p* < 0.0001; DBP 69.65 vs. 65.28 mmHg, *p* < 0.0001). Furthermore, a greater proportion of men reported smoking and alcohol consumption habits, whereas there were no significant differences in exercise habits between the sexes.

### Classification of adiposity indices for predicting new-onset hypertension

**Fig. 2:**
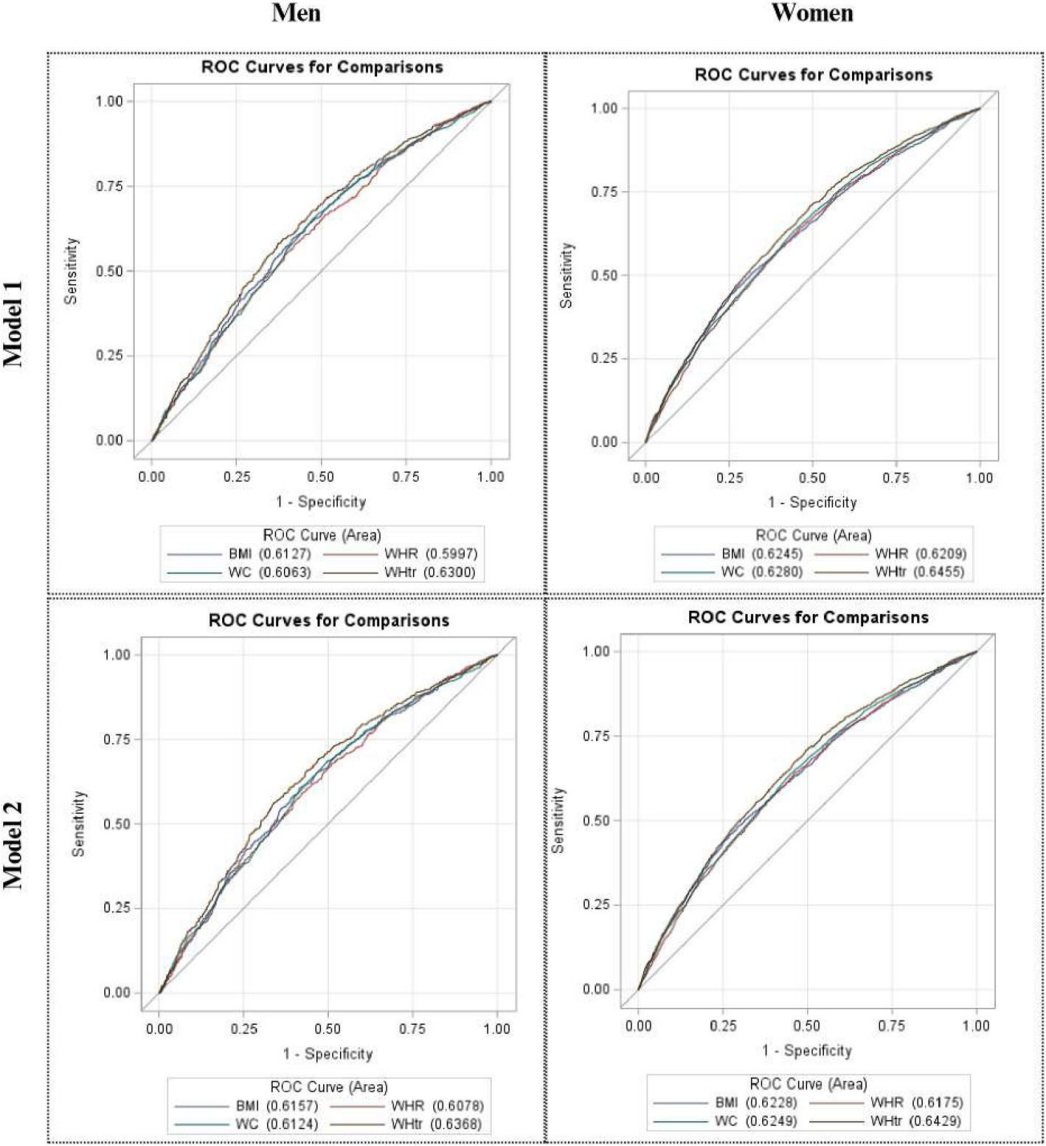
Receiving Operating Characteristics (ROC) of adiposity indices for new-onset hypertension in metabolically healthy adults. Model 1 was unadjusted; Model 2 was adjusted for age, total cholesterol, smoking status, alcohol consumption and menopausal status (for women). BMI, body mass index; WHR, waist-to-hip ratio; WC, waist circumference; WHtR, waist-to-height ratio

The receiver operating characteristic curves (ROCs) and area under the curves (AUCs) are shown in Fig. 2 and Table 2. In general, adiposity indices have demonstrated the ability to predict new-onset hypertension (AUC 0.58–0.66). Specifically, WHtR showed a higher AUC than the other indices among men and women tested using the Wald test. These differences remained significant even after adjusting for multiple covariates (Table 2, Model 2).

**Table 2:** Area under the curve (AUC) of adiposity indices for new-onset hypertension in metabolically healthy adults by sex

		**Men**		**Women**	
		AUC	95%CI	AUC	95%CI
**Model 1**	BMI	0.613^d^	0.591- 0.635	0.624^d^	0.609–0.640
	WHR	0.600^d^	0.577–0.622	0.621^d^	0.606–0.636
	WC	0.606^d^	0.584–0.629	0.628^d^	0.613–0.643
	WHtR	0.630^a, b, c^	0.608–0.652	0.646 ^a,b,c^	0.631–0.660
**Model 2**	BMI	0.616^d^	0.592–0.640	0.623^d^	0.607–0.638
	WHR	0.608^d^	0.584–0.632	0.618^d^	0.602–0.633
	WC	0.612^d^	0.588–0.637	0.625^d^	0.610–0.640
	WHtR	0.637 ^a, b, c^	0.613–0.661	0.643 ^a, b, c^	0.628–0.658

Model 1 was unadjusted; Model 2 was adjusted for age, total cholesterol, smoking status, alcohol consumption, and menopausal status (for women)

*BMI *Body mass index, *WHR *Waist-to-hip ratio, *WC *Waist circumference, *WHtR *Waist-to-height ratio, the letters a,b,c,d represent significant differences to each index

^a^significantly different with BMI (*p* < 0.05)

^b^significantly different with WHR (*p* < 0.05)

^c^significantly different with WC (*p* < 0.05)

^d^significantly different with WHtR (*p* < 0.05)

### Sensitivity, specificity and cutoff points of adiposity indices in metabolically healthy adults

Table 3 presents the cutoff points of the adiposity indices in both men and women. The WC and WHR’s cutoff points were higher in men than in women (WHR 0.87 vs. 0.82; WC 82 cm vs. 76.8 cm), whereas BMI (23.59 kg/m2 vs. 23.20 kg/m2) and WHtR (0.49 vs. 0.48) were similar in both sexes. The cutoff point of waist circumference (WC) in men demonstrated the highest sensitivity (67%), while the waist-to-hip ratio (WHtR) showed the highest specificity (63%). In addition, the WHtR cutoff point in women was associated with the highest sensitivity (71%), whereas body mass index (BMI) had the highest specificity (73%).

**Table 3:** The sex-specific cutoff point of adiposity indices for new-onset hypertension prediction in metabolically healthy adults

Indices	Sens	Spec	Max J	Cutoff
**Men**				
BMI	0.59	0.59	0.1811	23.59
WHR	0.58	0.58	0.1573	0.87
WC	0.67	0.51	0.1790	82
WHtR	0.58	0.63	0.2084	0.49
**Women**				
BMI	0.46	0.73	0.1907	23.20
WHR	0.64	0.54	0.1814	0.82
WC	0.63	0.56	0.1906	76.8
WHtR	0.71	0.51	0.2156	0.48

*AUC* Area Under the Curve, *CI* Confidence Interval, *Sens* Sensitivity, *Spec* Specificity, *Max J* Maximal Youden Index, *BMI* Body mass index, *WHR* Waist-to-hip ratio, *WC* Waist circumference, *WHtR* Waist-to-height ratio

The cutoff points for new-onset hypertension according to age in the metabolically healthy population are presented in Table S1 in the supplementary materials. According to age, WHR and WHtR showed similar cutoff points among age categories, whereas BMI had higher cutoff points in middle age and WC had higher cutoff points in young adults.

**Fig. 3:**
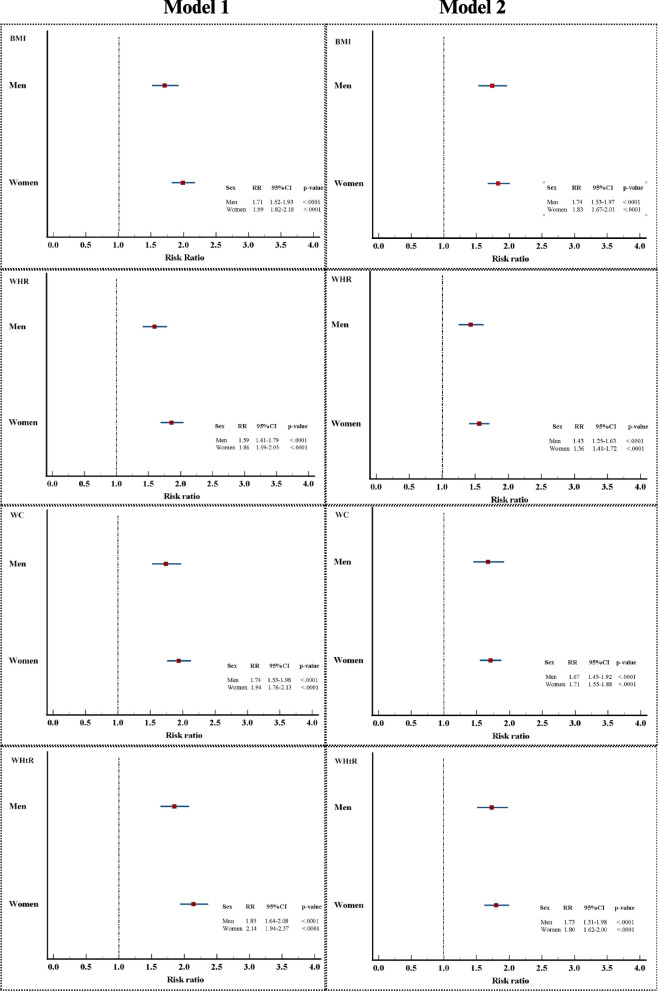
The sex-stratified new-onset hypertension risk in metabolically healthy adults determined using adiposity indices. Model 1 was unadjusted; Model 2 was adjusted for age, total cholesterol, smoking status, alcohol consumption and menopausal status (for women). BMI, body mass index; WHR, waist-to-hip ratio; WC, waist circumference; WHtR, waist-to-height ratio

The risk of new-onset hypertension was found to differ between individuals in the high and low categories based on sex-specific cutoff points. In particular, four adiposity indices revealed higher risks for women than men, although the differences were not statistically significant. However, the risk differences between men and women decreased in the adjusted model (Model 2) (see Fig. 3).

**Fig. 4:**
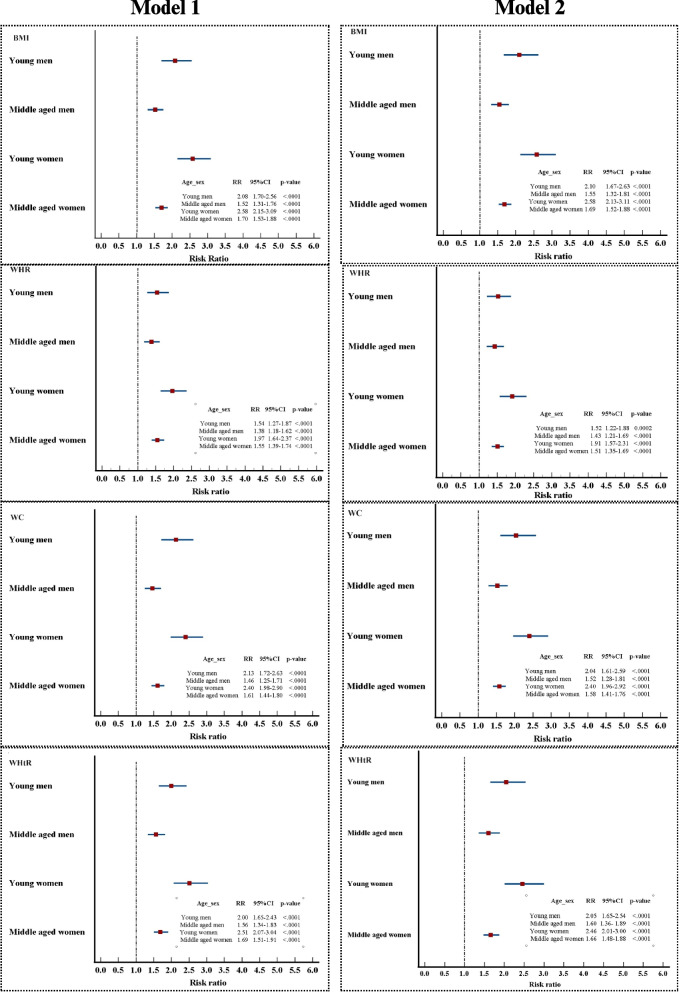
The age- and sex- stratified new-onset hypertension risk in metabolically healthy adult determined using adiposity indices. Model 1 was unadjusted; Model 2 was adjusted for age, total cholesterol, smoking status, alcohol consumption and menopausal status (for women). BMI, body mass index; WHR, waist-to-hip ratio; WC, waist circumference; WHtR, waist-to-height ratio

We extended our analysis to encompass age, examining the new-onset hypertension risk assessed by adiposity indices for both men and women in the young and middle-aged populations. Our research determined that young women had a significantly higher risk of developing new-onset hypertension when compared to middle-aged women when utilizing BMI, WC, and WHtR as adiposity indices (unadjusted: BMI RR = 2.58, 95%CI: 2.15–3.09, *p* < 0.0001 vs 1.70, 95%CI 1.53–1.88, *p* < 0.0001; WC RR = 2.40, 95%CI: 1.98–2.90, *p* < 0.0001 vs 1.61, 95%CI 1.44.-1.80, *p* < 0.0001; WHtR RR = 2.51, 95%CI: 2.07–3.04, *p* < 0.0001 vs 1.69, 95%CI 1.51–1.91,*p* < 0.0001; adjusted: BMI RR = 2.58, 95%CI: 2.13–3.11, *p* < 0.0001 vs 1.69, 95%CI 1.52–1.88,*p* < 0.0001; WC RR = 2.40, 95%CI: 1.96–2.92, *p* < 0.0001 vs 1.58, 95%CI 1.41–1.76, p < 0.0001; WHtR RR = 2.46, 95%CI: 2.01–3.00, *p* < 0.0001 vs 1.66, 95%CI 1.48–1.88, *p* < 0.0001). In the unadjusted model utilizing waist circumference (WC), a substantial increase in the risk of hypertension was observed among young men compared with middle-aged men (young men RR = 2.13, 95%CI: 1.72–2.63, *p* < 0.0001 vs middle-aged men RR = 1.46, 95%CI: 1.25–1.71, *p* < 0.0001). Additionally, for other adiposity indices, no significant differences in risk were observed between young and middle-aged men. When comparing the various age categories, young and middle-aged women exhibited a similar risk of hypertension compared to their respective male counterparts, as depicted in Fig. 4.

## Discussion

This prospective cohort study highlighted the effectiveness of adiposity indices (BMI, WHR, WC, and WHtR) for independently predicting new-onset hypertension in metabolically healthy adults in both unadjusted and adjusted models. Although there is still controversy surrounding which adiposity indices have the best performance for predicting new-onset hypertension, our findings showed that the WHtR had the highest prediction performance among adiposity indices in both sexes, which is consistent with several previous studies. Lin et al*.* reported that the WHtR was an ideal indicator for screening cardiovascular disease risk factors, particularly for hypertension; the AUCs of the WHtR were 0.658 for men and 0.752 for women [32].

A systematic review and meta-analysis of the potential screening ability of the WHtR reported that the WHtR had a higher discriminative power for cardiometabolic risk (4%–5%) than BMI and WC did.[33] Choi JR. et al*.* reported that WHtR improved hypertension prediction of age, sex, and SBP (AUC age + sex + SBP 0.675; AUC age + sex SBP + WHtRR 0.722, *p* = 0.001) [34]. Since WHtR was one of the parameter reflecting central adiposity, this result also emphasized stronger effect of central adiposity toward hypertension [35, 36]. The potential explanation on how including an individual’s stature when analyzing central adiposity can improve its discriminative power for hypertension was because of the effects of stature on vascular function and fat distribution [37, 38]. Moreover, short stature is associated with higher central adiposity and lower vasculature diameter resulting in high blood pressure level [33, 37, 38].

The optimal sex cutoff points for predicting new-onset hypertension in our study were calculated using the maximal Youden Index method. The cutoff points for BMI were 23.59 kg/m^2^ for men and 23.20 kg/m^2^ for women. In the previous study, the BMI cutoff points for hypertension were 23.9 kg/m2 for men and 22.5 kg/m2 for women [32]. WHO (World Health Organization) also set a 23 kg/m^2^ threshold to predict hypertension in the Asia Pacific population [39]. The cutoff points for WHR in this study were 0.87 for men and 0.82 for women, comparable with a similar study conducted using the Taiwanese population, 0.86 for men and 0.77 for women [32]. The WC cutoff point for men was 82 cm, and for women, it was 76 cm. The threshold was similar for men compared to the previous study using the Taiwanese population, which was 81.5 cm, and higher in women compared to the same study, which was 72.5 cm [32]. The WHtR cutoff points were similar between men (0.49) and women (0.48). This finding was similar to a prior study, which had cutoff points of 0.48 for men and 0.46 for women, and also other kinds of literature that set up a threshold for WHtR in 0.5 for both sexes to predict cardiometabolic disease, including hypertension [32, 34, 40, 41].

Our study observed that the cutoff points for BMI, WC, and WHR among men were comparable to those of the general Taiwanese population. However, the cutoff points for women were higher for all three adiposity indices. These results add to the evidence suggesting a greater impact of metabolic status on women than men. Additionally, our study verified the consistency of the WHtR cutoff point (0.5) for hypertension prediction in metabolically healthy Taiwanese adults since the cutoff points remain stable among sexes and age categories.

In this study, men had a higher incidence of new-onset hypertension than women did. However, prediction using sex-specific cutoff points of adiposity indices carried a similar risk of new-onset hypertension in both sexes, even after multivariable adjustment. This finding underlined that the burden of excess adiposity in women was equal to that in men, and sex-specific factors played an important role in the mechanism of adiposity-related hypertension. Gender, sociocultural, environmental, and psychological factors have complex interactions underlying the differential impact of excess adiposity between the sexes [42]. Besides sex hormones, women's sedentary lifestyle, unhealthy diet, and stress increase the impact of excess adiposity, especially on cardiometabolic diseases [43].

When stratified by sex and age, young women with excess adiposity had a significantly higher risk of hypertension than middle-aged women. Previous research has confirmed that young adults have the highest risk of weight gain and metabolic consequences [44–46]. Among cardiometabolic risk factors, high WC and high BMI had a stronger link to increased high sensitivity C-reactive protein (hs-CRP) in young adults [46]. According to the results of our study, young women, who are believed to be protected by estrogen, are at a higher risk of developing new-onset hypertension than middle-aged women, and their risk is comparable to that of young men.

Previous reports have suggested that in younger adults, men have higher blood pressure than women and that women’s blood pressure tends to increase more rapidly during midlife, eventually surpassing that of men [47–49]. These phenomena observed indicate that the protective effects of estrogen decrease during midlife [50]. However, this new information contradicts those reports and states the opposite. Therefore, it is essential to carefully consider the new-onset hypertension risk faced by young women. One possible explanation for the higher risk of new-onset hypertension in young women is alteration of sex-hormone regulation and protective effect by excessive and malfunctioning adipose tissue, as predicated by adiposity indices [51, 52]. It has been suggested that early menarche in women may be linked to excess body fat in young women [53, 54]. Nonetheless, the exact mechanism by which excess adiposity leads to new-onset hypertension is complex and involves multiple factors. Further research is needed to identify the specific pathological pathways involved in adiposity-related hypertension in women.

Our study has several strengths, including the use of a large prospective cohort and adequate follow-up. This is the first study to investigate the predictive performance of adiposity indices for new-onset hypertension in metabolically healthy Taiwanese adults. Despite these strengths, our study had some limitations. First, we did not collect data regarding therapy for cardiometabolic diseases, which may have affected our results. Second, our study lacked exact data on antihypertensive therapy and drug use and may have been influenced by recall bias due to our inclusion of patients with self-reported hypertension. Finally, our study used office blood pressure measurements, meaning masked hypertension may not have been detected.

In summary, a high level of adiposity index, as determined by sex-specific cutoff points, indicates a higher risk of new-onset hypertension in metabolically healthy Taiwanese adults. It is worth noting that young women should be a particular concern as they have a significantly greater risk of new-onset hypertension compared to middle-aged adults, and their risk is comparable to that of young men.

### Supplementary Information

**Supplementary Material 1.**

## Data Availability

The dataset used in the current study is available at the request of the corresponding author (baich@tmu.edu.tw).

## Abbreviations

BMI: Body mass index
WHR: Waist-to-hip ratio
WC: Waist circumference
WHtR: Waist-to-height ratio
FPG: Fasting plasma glucose
TG: Triglyceride
TC: Total cholesterol
HDL-C: High-density lipoprotein cholesterol
ROC: Receiver operating characteristic
AUC: Area under the curve
SBP: Systolic blood pressure
DBP: Diastolic blood pressure
TWB: Taiwan Biobank
