# Supplementary material for: Adiposity indices and their higher predictive value for new-onset hypertension in metabolically healthy young women: findings from a population-based prospective cohort study
Source: BMC Cardiovasc Disord. 2024 Mar 12;24:150. doi: 10.1186/s12872-024-03817-y (PMC10935983; doi:10.1186/s12872-024-03817-y)
Supplement: Supplementary file 1 — Supplementary Material 1. [file 12872_2024_3817_MOESM1_ESM.pdf]

Table S1. Cut-off point of new onset hypertension according to age.

Adiposity indices	Young adult (20- 45-year-old)				Middle age (>45 – 65-year-old)			
	Sens	Spec	Max J	Cut-off	Sens	Spec	Max J	Cut-off
BMI	0.62	0.66	0.2823	22.82	0.50	0.67	0.1770	23.18
WHR	0.69	0.54	0.2289	0.82	0.65	0.51	0.1595	0.84
WC	0.67	0.61	0.2820	78.1	0.70	0.46	0.1578	76.8
WHtR	0.72	0.54	0.2589	0.47	0.52	0.64	0.1593	0.50

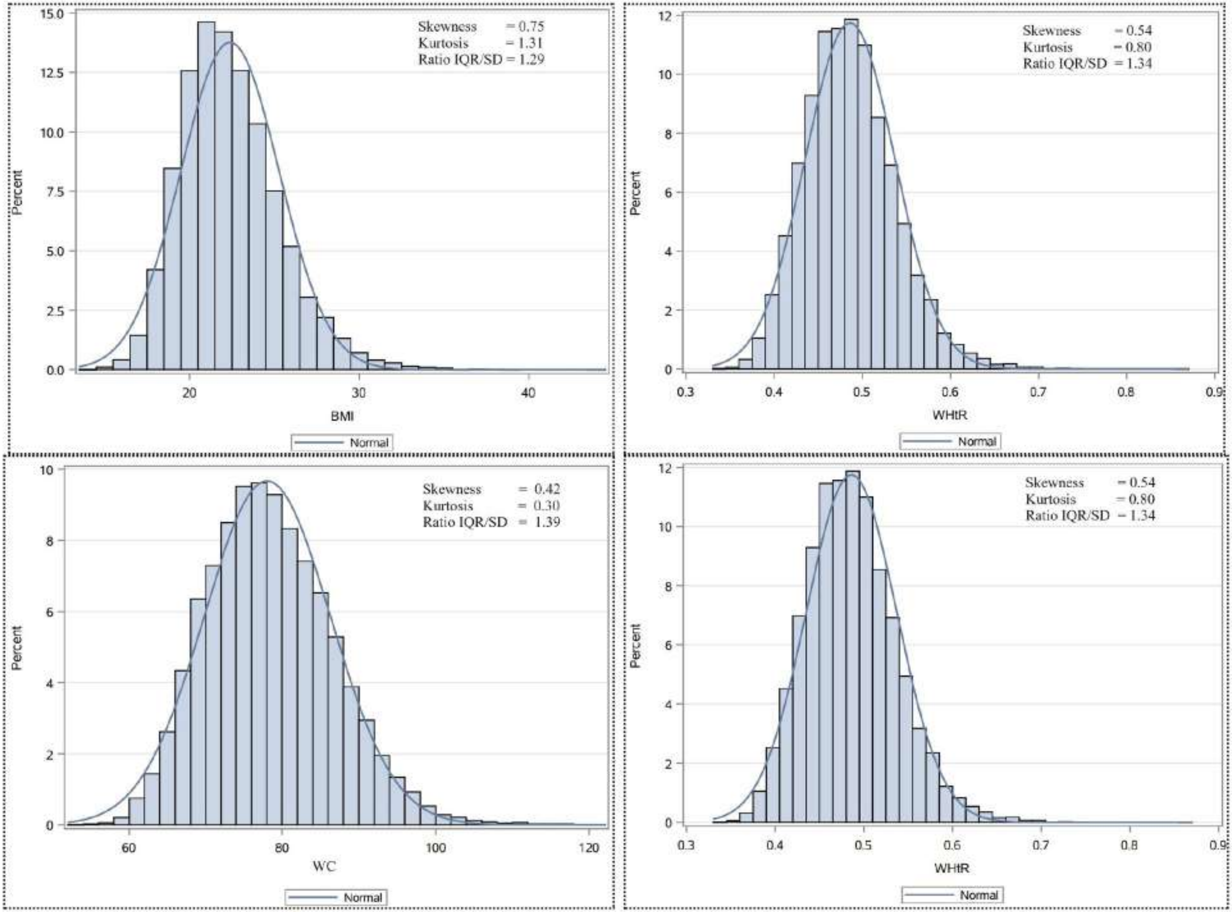

Figure S1. Histogram and distribution of adiposity indices; BMI, body mass index; WHR, waist-to-hip ratio; WC, waist circumference; WHtR, waist-to-height ratio; IQR, interquartile range; SD, standard deviation.
